# Modeling of CO_2_ adsorption capacity by porous metal organic frameworks using advanced decision tree-based models

**DOI:** 10.1038/s41598-021-04168-w

**Published:** 2021-12-28

**Authors:** Jafar Abdi, Fahimeh Hadavimoghaddam, Masoud Hadipoor, Abdolhossein Hemmati-Sarapardeh

**Affiliations:** 1 Faculty of Chemical and Materials Engineering, Shahrood University of Technology, 3619995161 Shahrood, Iran; 2 Ufa State Petroleum Technological University, Ufa, Russia 450064; 3 Department of Petroleum Engineering, Ahwaz Faculty of Petroleum Engineering, Petroleum University of Technology (PUT), Ahwaz, Iran; 4 Department of Petroleum Engineering, Shahid Bahonar University of Kerman, Kerman, Iran; 5 College of Construction Engineering, Jilin University, Changchun, 130600 China

**Keywords:** Environmental sciences, Energy science and technology, Engineering

## Abstract

In recent years, metal organic frameworks (MOFs) have been distinguished as a very promising and efficient group of materials which can be used in carbon capture and storage (CCS) projects. In the present study, the potential ability of modern and powerful decision tree-based methods such as Categorical Boosting (CatBoost), Light Gradient Boosting Machine (LightGBM), Extreme Gradient Boosting (XGBoost), and Random Forest (RF) was investigated to predict carbon dioxide adsorption by 19 different MOFs. Reviewing the literature, a comprehensive databank was gathered including 1191 data points related to the adsorption capacity of different MOFs in various conditions. The inputs of the implemented models were selected as temperature (K), pressure (bar), specific surface area (m^2^/g) and pore volume (cm^3^/g) of the MOFs and the output was CO_2_ uptake capacity (mmol/g). Root mean square error (RMSE) values of 0.5682, 1.5712, 1.0853, and 1.9667 were obtained for XGBoost, CatBoost, LightGBM, and RF models, respectively. The sensitivity analysis showed that among all investigated parameters, only the temperature negatively impacts the CO_2_ adsorption capacity and the pressure and specific surface area of the MOFs had the most significant effects. Among all implemented models, the XGBoost was found to be the most trustable model. Moreover, this model showed well-fitting with experimental data in comparison with different isotherm models. The accurate prediction of CO_2_ adsorption capacity by MOFs using the XGBoost approach confirmed that it is capable of handling a wide range of data, cost-efficient and straightforward to apply in environmental applications.

## Introduction

Carbon dioxide (CO_2_) plays a high influencing role in global warming^1^. As a result of consumption of fossil fuels especially in electricity generation, transportation and other industrial activities, CO_2_ emission into the atmosphere is surging^2,3^. According to an investigation by Pachauri et al. in 2014, carbon dioxide’s concentration in the atmosphere has increased from 280 to 400 ppm with 0.8 °C^1,4^. It is estimated that concentration of CO_2_ would touch a peak of 600–700 ppm at the dawn of 22th century and resultantly it will lead in 4.5–5 °C growth in the average temperature of earth^5^. Thus, as to put a halt on rapid growth of CO_2_ emission rates, United States Department of Energy (DOE) provided the world with a program aiming for reduction of CO_2_ concentrations by utilization of high efficiency CO_2_ capture plans. According to the issued program 90% of emitted CO_2_ could be captured just with less than 35% of additional budget allocation to carbon capture and storage (CCS) programs^6^. These programs could be contemplated as promising approaches for the separation and sequestration of CO_2_. As soon as carbon dioxide is separated, it could be stored underground, or alternatively it can be utilized in various industries such as oil industries in order to enhance the recovery^7–9^. Up to date, scientists have proposed numerous methods for CO_2_ capture among which absorption^10–12^, membranes^13,14^, and carbon-based adsorbents^5,13^ are well developed. Nevertheless; high total costs, low capacities, and challenging regeneration processes are some of their limitations^15^. A material which could be used successfully for an efficient CO_2_ capture process, not only should possess good characteristics for CO_2_ uptake, but also must release the captured carbon dioxide in the regeneration step. Furthermore, the effectiveness of the capturing process could be maximized provided that the structure of the material could be modified using various functional groups and molecular tuning approaches.

Metal organic frameworks (MOFs) have been distinguished as a very promising and efficient group of materials which can be used in CCS projects because of their unique properties of being modifiable, stable in high temperatures, and having a chemical structure which can be easily adjusted^16–20^. Therefore, different advantages and disadvantages of MOFs have been investigated by several researchers such as an investigation done by Le et al., which reported how the architecture and active functional groups of MOFs could be controlled^17^. Moreover, having conducted various investigations on the thermal stability of MOFs, scientists have reported the impact of morphology and crystalline shape of these materials on their thermal stability^21–24^. Taking MOFs synthesized from strontium and calcium by the way of example, Yeh et al.^25^ found that, as a result of their micro-porosity, these materials were thermally stable in all temperatures lower than 450 °C. Some other scientific investigations have found that MOFs could be modified for various applications just by changing the functional groups, which are located on their pore walls^26,27^. It is wieldy known that MOFs are comprised of metal ions clusters and linkers which are organic molecules. These linkers have a 3D-structure of pores and connecting channels. While 3D-structurevoids adsorb molecules as their host, the primary structure of these materials provides reversible channels and pores as soon as desorption step take place^26–28^. Properties of MOFs is determined by the selected linker and the metal. For example, zinc in the structure of IRMOF-1 is the metal which is located at the center of structures and is connected to the terephthalic molecules. Having this structure, IRMOF-1 benefits from existence of pores with high capacity of adsorption. Furthermore, there are various groups of MOFs with their unique structures, properties and application. Some MOFs like UMCs have unsaturated metallic centers^29^, which provides carbon dioxide molecules with more active sites, hence facilitates formation of strong bonds between CO_2_ and the structure. Other advantage of MOFs in comparison to other materials like zeolite is that the MOFs have typically wider pores, which boost diffusion rate of molecules not only in a single structure, but also between different crystals^30–33^. That is why scientists believe that MOFs are promising for adsorption of CO_2_, emphasizing on their adjustable porosities and having a modifiable surface chemistry^5,34–36^. Regarding developing and testing MOF, there are some important challenges and obstacles to overcome or be solved using alternative methods. Firstly, experimental investigation of adsorption capacity of MOFs not only is time consuming, but also is not cost-effective. Secondly, data cannot be matched with the developed isotherms because typically these isotherms are proposed for a specific range of data^31^. So as to address the problem, many scientists have been trying to use soft computing methods by which not only the time and money could be saved, but also there is no need for simplifying assumptions and the data could be modeled more precisely. Artificial intelligence (AI) approaches are useful tools which enable us to estimate and develop representative models in various disciplines^37–42^. These powerful algorithms are able to model non-linear relationship which exist between influencing parameters. Up to the date, a plethora of models have been developed such as fuzzy logic, radial basis networks, support vector machine, and colony optimization^43–47^.

In the current investigation, authors tried utilizing smart models for the prediction of nonlinear adsorption of CO_2_ by MOFs. According to the literature, scientists have done fewer researches on the modeling of CO_2_ adsorption by MOFs using AI methods. To model the CO_2_ uptake capacity of different MOFs, new and powerful methods of CatBoost, LightGBM, Random Forest (RF), and XGBoost were employed. CatBoost (which is the abbreviated form of categorical boosting) is an open-source and modern gradient boosting library and it can deal with problems which are intrinsically heterogeneous thought handling categorical features. XGboost and LightGBM belong to GBDTs (Gradient Boosted Decision Trees) and in these methods a tree structure includes two separate steps. Firstly, the appropriate structure for the tree must be found. Secondly, leaf values must be set as soon as the tree structure is finalized^48^. Another approach which was used in this investigation was Random Forest. Since it was introduced, the accuracy of classifications improved significantly because not only growth of various trees were allowed, but also the program is able to vote for best and most distinguished class^49^. Reviewing literature, the authors gathered 1191 data points related to the adsorption capacity of different MOFs at various temperatures and pressures. The investigated MOFs are ZIF-8, Zn-MOF-74, Mg-MOF-74, PCN-16, MOF-5, PCN-11, BeBTB, Co-BDP, Mg_2_(dobdc), Cu-BTTri, MOF-177, IRMOF-1, IRMOF-6, IRMOF-3, IRMOF-11, Cu-BTC, MOF-505, MOF-74, and MOF-2^50–53^.

## Implementation of models

### Extreme gradient boosting model (XGBoost)

**Figure 1:**
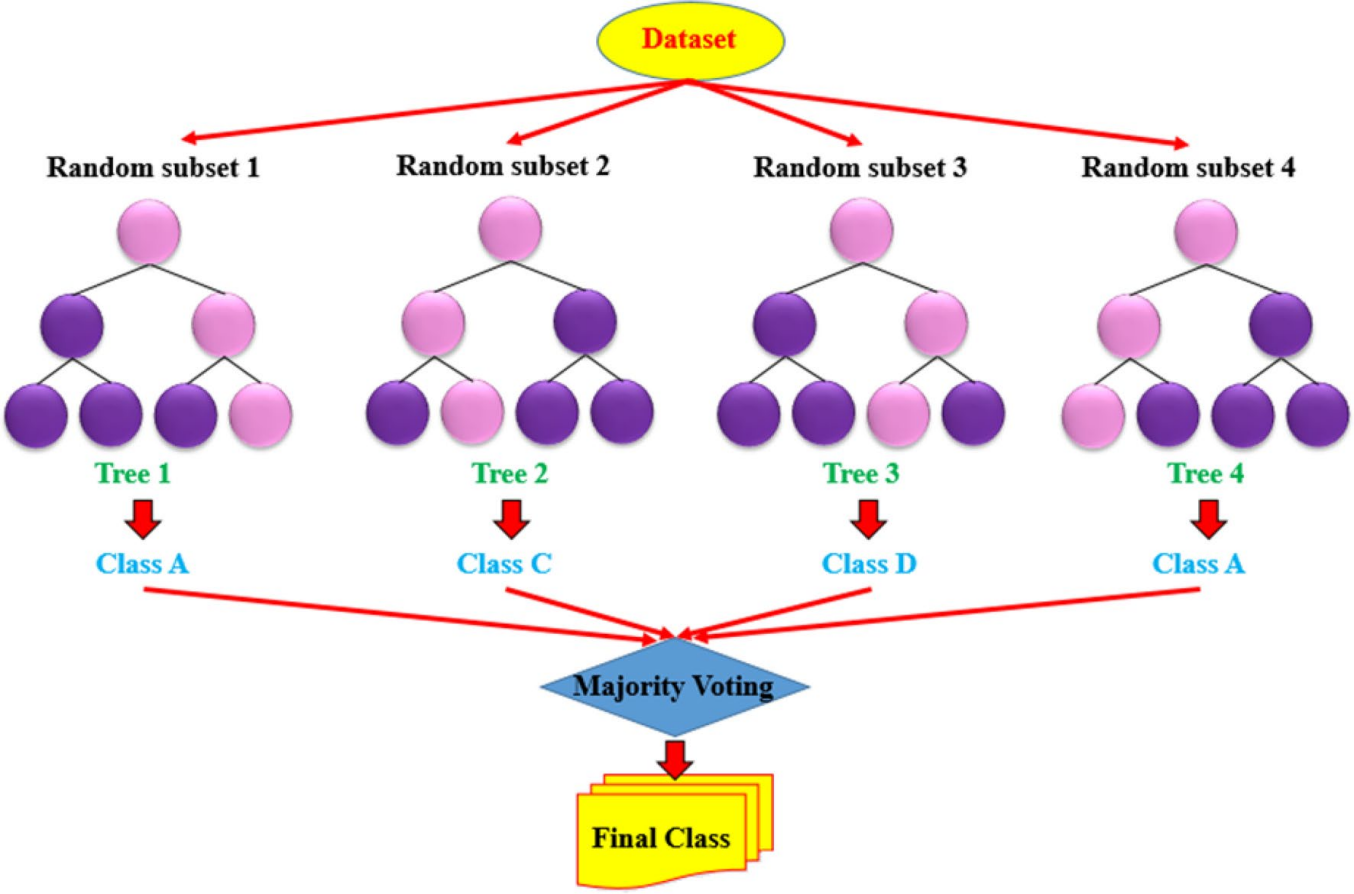
A schematic illustration of XGBoost depicting the main node, interior nodes, and the leaves.

In a tree-based ensemble method, a group of various classification and regression trees (CARTs) are utilized to minimize a set of objective functions applied to a training dataset. The XGBoost approach could be contemplated as a tree-based model which basically belongs to a gradient boosting decision tree (GBDT). So as to explain the CART’s basic structure, it is comprised of three various nodes namely (a) the main node (root node), (b) internal nodes, and (c) leaf nodes like as illustrated in Fig. 1. The binary decision-making processes will split the root node into internal nodes. Doing so, the dataset which is located in the root will be classified into various nodes in the internal nodes and the final classification will take place in the leaf nodes as the final classes. Aiming for developing a powerful set according to the gradient booting model, an ensemble of CATRs are introduced and developed using determination of their influence by giving them a specific weight during the training process^54^.

In a dataset where m dimension features and n examples exist, the modeling output (y) would be trained according to the following expression to form n tree nodes^55^:

$$\hat{y}_{i} = \mathop \sum \limits_{k = 1}^{N} f_{k} \left( {X_{i} } \right), \quad f_{k} \in f$$

1 $$where \; f = \left\{ {f\left( X \right) = \omega_{q\left( x \right)} } \right\}, \left( {q:m \to T, \omega \in T} \right)$$

where a binary leaf index will be formed by mapping an example X using a defined decision rule *q(x)*. In Eqs. (1) and (2), the corresponding space of each regression tree is depicted by ‘*f’*. Accordingly, *f*_*k*_ represents the kth tree, tree leaves are denoted by *T*, and their corresponding weight is determined by *ω*.

As the next step in the modeling, tree sets will be determined by minimizing an objective function denoted by *L*^55^:

$$L = \mathop \sum \limits_{i = 1}^{n} l(\hat{y}_{i} ,y_{i} ) + \mathop \sum \limits_{k = 1}^{N} {\Omega }(f_{k} )$$

2 $$with\, {\Omega }\left( f \right) = \gamma T + \frac{1}{2} \lambda \omega ^{2}$$

In the given formulation, *Ω* represents the regularization function and limits the model complexity by reducing the overfitting issues; loss function is shown by *l* and intrinsically is a differentiable convex; the minimum loss is denoted by *γ* and it is necessary in division of a new ultimate class as a leaf, and *λ* stands for the regulation coefficient. *γ* and *λ* facilitates the growth of the variance of the model and resultantly plummet the overfitting issue^55^. Every leaf in the boosting model has its own objective function, which should be minimized iteratively as follows^55^:

3 $$L^{\left( t \right)} = \mathop \sum \limits_{i = 1}^{n} \left\{ {l\left( {y_{i} ,\hat{y}_{i}^{{\left( {t - 1} \right)}} } \right) + f_{t} \left( {X_{i} } \right)} \right\} + {\Omega }\left( {f_{t} } \right)$$

In the presented formula, *t* is the iteration number for the minimization of a leaf objective function in the training step. So as to improve the model, an algorithm known as the greedy algorithm is utilized, which is designed to provide enough space for regression trees. Doing so, XGBoost model can continuously update its final results through improving the preciseness of the objective functions^55^:

4 $$\hat{y}_{i}^{\left( t \right)} = \hat{y}_{i}^{{\left( {t - 1} \right)}} + f_{t} \left( {X_{i} } \right)$$

Shrinkage strategy is another strategy that XGBoost method utilizes properly. In this strategy, a learning factor is defined and its learning rate is regulated in every gradient boosting step through definition of additional weights. Shrinkage strategy prevents overfitting problem by restricting future trees from affecting previously formed trees^56^.

### Light gradient boosting machine (LightGBM)

On the basis of gradient learning theories, a novel learning machine was developed which is known as LightGBM^57^. The LightGBM approach, in comparison to XGBoost, needs lower memory spaces and speeds up the training step by using a histogram^58^. LightGBM can form a histogram with a width of ‘k’ by discretizing eigenvalues into ‘k’ different bins. Furthermore, the aforementioned approach diminishes the need for a set of pre-sorted results and values will be saved in an integer with a size of eight bits, which results in a drastic reduction of memory consumption. That said, such as kind of approach unfortunately results in dipping the model’s preciseness. Leaf-wise approach has also been utilized in LightGBM. Drawing comparison between traditional growth strategies and the leaf-wise strategy, it must be admitted that this approach is considerably more efficient than the others. What makes the leaf-wise strategy more efficient than the alternative level-wise strategy is this fact that the leaves existing in the same layer are properly taken into consideration, which diminish the need for unnecessary allocation of memory. Therefore, by finding leaves with the maximum branching gain, errors could be minimized and a better accurateness could be achieved. In Fig. S1, the leaf-wise strategy of tree development is illustrated. An important drawback of leaf orientation is that as the decision trees grow deeper, unfavorable overfitting gets exacerbated. That said, simultaneously when LightGBM is resulting in overfitting, by definition of an upper limit on depth of the leaf top, a high efficiency will be achieved^57,58^.

Regarding the formulation of a LightGBM model, parameters and calculations could be introduced as follows^59^:

Being given a training dataset of $X = \left\{ {x_{i} ,y_{i} } \right\}_{i = 1}^{m}$, the LightGBM approach approximates a $\hat{f}\left( x \right)$ according to a $f^{*} \left( x \right)$ aiming for the minimization of the desirable values of a loss function shown by $L\left( {y,f\left( x \right)} \right){:}$

5 $$\hat{f}\left( x \right) = \arg \mathop {\min }\limits_{f} E_{y,x} L\left( {y,f\left( x \right)} \right)$$

A wide variety of *T* regression trees with the formulation of $\mathop \sum \limits_{t = 1}^{T} f_{t } \left( x \right)$ will be formed as LightGBM sets which can be used for the model’s approximation. In a defined regression tree ($W_{q\left( x \right)} , q \in \left\{ {1,2, \ldots ,N} \right\}$), *w* stands for a vector which represents the weight of each leaf node, *N* depicts how many leaves exist in a tree, and the decision rules applied to trees are shown by *q*. The training step of the model development, at step t, is formulated as follows^59^:

6 $$G_{t} \cong \mathop \sum \limits_{i = 1}^{N} L\left( {y_{i} ,F_{t - 1} \left( {x_{i} } \right) + f_{t} \left( {x_{i} } \right)} \right)$$

The objective functions are determined by utilizing Newton's method.

### Gradient boosting with categorical features support (CatBoost)

In the CatBoost approach aiming for successful application of categorical boosting technique, categorical columns must be utilized. The aforementioned column benefit from a range of processing techniques. The target-based statistics and the one_hot_max_size (OHMS) are the most important ones. In the growth of every branch of the running tree, a greedy approach will be applied to facilitate finding changes in the combination of features of CatBoost method^60^. In the CatBoost method, the following steps must progress properly for every feature of various categories^48,61^:

1. To form a random subgroup of the available records
2. Convert labels to integer
3. According to Eq. (7), features related to categories must be transformed into numeric form:
  7 $$avgT arg et = \frac{countInClass + prior}{{totalCount + 1}}$$

In the given formulation, targets are counted by *countInClass.* Each target is assigned with some categorical features, each having a value of one, and all the previous objects will be counted in *totalCount* (the *prior* ones which are needed in counting the objects are determined by the initiating parameters)^48,61^.

### Random forest (RF)

An ensemble of various decision trees forms a random forest, in which trees are being trained in parallel. The superiority and the importance of each decision tree is determined by the algorithm^62^. Additionally, a built-in property of RF classifier which is utilized to select various features makes the RF able to manage different inputted features without need for removing a number of parameters for reduction of dimension^63^. In the modeling, in order to enhance the diversity of trees of the forest, the RF technique employs a method known as Bagging (which stands for bootstrap aggregating). Typically, the population of trees are given as an input to the model, and accordingly the model will divide data points into various sets. Being a type of random sampling methods, bagging employs just a third of data points for the training step of a subtree development process and the remaining data points are referred as the out-of-bag (OOB). Furthermore, in the model development cross-validation of results are not required in the RF, as the accuracy of the model is assessable using the OOB’s errors^49^. In Fig. S2, the strategy of RF method is illustrated. A successful training process will happen if a training sample is given to the model as a requirement. If there is a training data set in the form of $D = \left[ {\left( {x_{1} .y_{1} } \right).\left( {x_{2} .y_{2} } \right). \cdots \left( {x_{n} .y_{n} } \right)} \right]$, the defined training dataset for tree $h_{t}$ will be denoted by $D_{t}$, and the resulting prediction of the out-of-bag dataset of sample x will be $H^{oob}$, as it is given as follows^49^:

8 $$H^{oob} \left( x \right) = argmax\mathop \sum \limits_{t = 1}^{T} I\left( {h_{t} \left( x \right)} \right) = y$$

For the purpose of the modeling, the error of the OOB dataset is generalized as follows^49^:

9 $$\varepsilon^{oob} \left( x \right) = \frac{1}{\left| D \right|}\mathop \sum \limits_{{\left( {x.y} \right)D}} I\left( {H^{oob} \left( x \right) \ne y} \right)$$

The RF’s operation should be random and this feature is controlled by a parameter formulated as $k = log_{2} d$^49^. The significance of a characteristic of a variable *X*_*i*_ could be measured using the following expression^49^:

10 $$I\left( {X_{i} } \right) = \frac{1}{B}\mathop \sum \limits_{t}^{B} \widetilde{OOBe}rr_{{t^{i} }} - OOBerr_{t}$$

Accordingly, in the X vector, the *i*^th^ factor is denoted by $X_{i}$, *B* depicts how many trees exist in the current RF, the predicted error of the OOB samples is defined by $\widetilde{OOB}err_{{t^{i} }}$, which stands for the feature $X_{i}$ of tree $t$, and finally the initial OOB data samples are given as the $OOBerr_{t}$, which includes the permuted variables.

**Figure 2:**
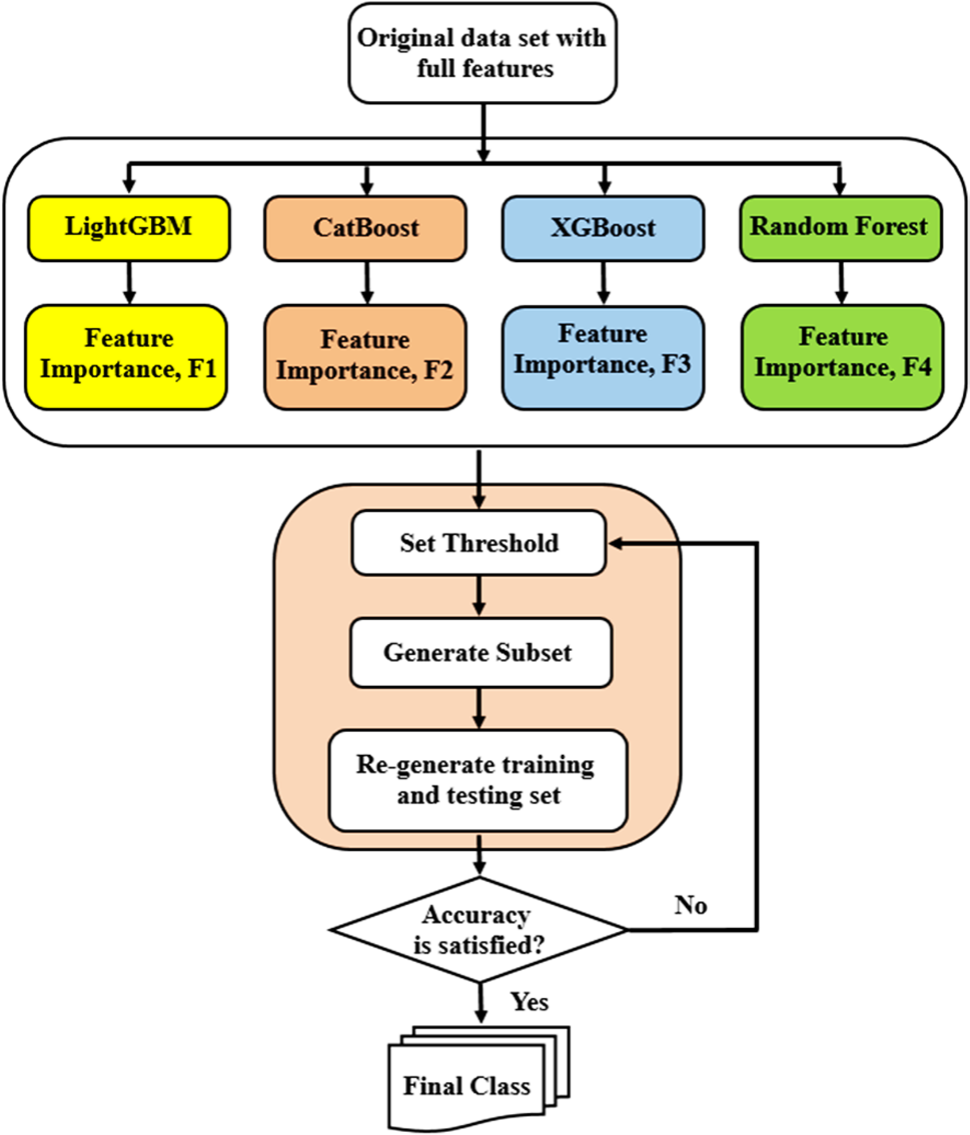
Feature selection and classification using different algorithm for predicting CO_2_ adsorption on MOFs.

The significance of the character permutation process illustrates how much a feature is salient for the estimation. Resultantly characteristic permutation severely changes the model’s estimation and it can be conclusively observed that an insignificant feature possess a very limited power for changing the prediction of the model^64^. Figure 2 shows the feature selection and classification using different algorithm for predicting CO_2_ adsorption on MOFs.

## Data gathering

A dataset comprising of approximately 1191 experimental results of CO_2_ uptake on various MOFs including ZIF-8, Zn-MOF-74, Mg-MOF-74, PCN-16, MOF-5, PCN-11, BeBTB, Co-BDP, Mg_2_(dobdc), Cu-BTTri, MOF-177, IRMOF-1, IRMOF-6, IRMOF-3, IRMOF-11, Cu-BTC, MOF-505, MOF-74, and MOF-2 was collected reviewing the literature^51–53^. Table 1 represents the details of the data used in this study. Moreover, Table 2 lists down the statistical details of the whole dataset which was collected for the purpose of this investigation. Trying to conceptualize and perceive the effect of various parameters on the MOF’s CO_2_ uptake capacity, the authors have incorporated temperature (K), pressure (bar), surface area (m^2^/g) and pore volume (cm^3^/g). To provide a precise and reliable set of models, deliberately about 80% of data points were devoted to model establishment and training phase and just about 20% were considered for testing phase. Therefore, the models’ preciseness and trustworthiness were ensured by utilizing two statistical factors, namely root mean-square error (RMSE) and coefficient of determination (R^2^) were used^65–67^:

11 $$RMSE = \left( {\frac{1}{N}\mathop \sum \limits_{i = 1}^{N} \left( {y_{i}^{estimated} - y_{i}^{exp.} } \right)^{2} } \right)^{0.5}$$

12 $$R^{2} = 1 - \frac{{\mathop \sum \nolimits_{i = 1}^{N} \left( {y_{i}^{estimated} - y_{i}^{exp.} } \right)^{2} }}{{\mathop \sum \nolimits_{i = 1}^{N} \left( {y_{i}^{etimated} - \overline{y}^{exp.} } \right)^{2} }}$$

**Table 1:** Details of the experimental data used in this study.

No	MOF	Surface area (m^2^/g)	Pore volume (cm^3^/g)	Temperature (K)	Pressure (bar)	CO_2_ uptake (mmol/g)	No. data	Refs.
1	MOF-2	345	0.227	298	0–42.2	0–3.20	39	^51^
2	MOF-74	816	0.4	298	0–42.4	0−10.4	31	^51^
3	MOF-505	1547	1.83	298	0–42.5	0–10.4	31	^51^
4	Cu_3_(BTC)_2_	1781	0.43	298	0–42.4	0−10.7	30	^51^
5	IRMOF-11	2096	0.92	298	0–42.4	0–14.8	32	^51^
6	IRMOF-3	2160	1.07	298	0–42.2	0–18.9	37	^51^
7	IRMOF-6	2516	1.14	298	0–42.5	0–19.7	37	^51^
8	IRMOF-1	2833	0.18	298	0–42.2	0–22.0	35	^51^
9	MOF-177	4508	1.59	298	0–42.5	0–33.9	70	^52^
10	CuBTTri	1750	0.713	313	0.546–39.86	1.16–16.99	43	^52^
11	Mg_2_(dobdc)	1800	0.5727	313	0.0005–35.32	0.09–15.15	51	^52^
12	CoBDP	2030	0.93	313	1.31–39.87	0.28–16.56	31	^52^
13	BeBTB	4030	1.701	313	1.02–38.83	1.79–30.17	40	^52^
14	PCN-11	1931	0.91	220–310	0–30.80	0 -22.84	104	^53^
15	MOF-5	3500	1.31	220–310	0–31.43	0–30.34	96	^53^
16	PCN-16	2273	1.06	220–310	0–31.66	0–21.51	120	^53^
17	HKUST-1	1690	0.66	220–310	0–24.99	0–19.36	130	^53^
18	Mg-MOF-74	1332	0.61	280–310	0–31.63	0–13.55	63	^53^
19	Zn-MOF-74	885	0.41	280–310	0–31.39	0–10.65	63	^53^
20	ZIF-8	1980	0.65	220–310	0–31.6	0–11.83	108	^53^
	Total			220–313	0–42.5	0–33.9	1191

**Table 2:** Statistical details of the dataset gathered in this paper.

	Pressure (bar)	Temperature (K)	Surface area: S (m^2^/g)	Pore volume: V_P_ (cm^3^/g)	CO_2_ uptake (mmol/g)
Mean	10.469	286.319	2156.937	0.877	10.910
STD	11.539	28.238	969.463	0.409	8.512
Min	0	220	345	0.180	0
25%	1	280	1690	0.610	4.9
50%	5.2	298	1980	0.910	9.899
75%	17.714	310	2273	1.070	16.065
Max	42.5	313	4508	1.830	141

### Outlier detection

Having a range of uncertainties and being associated with outliers, experimental data can introduce errors in the modeling process. To prevent any undesirable and not reliable outcome, the experimental models must undergo data evaluation and outlier detection. For the case of CO_2_ adsorption on various MOFs, the faulty data points will enormously impact the preciseness of the predicting models. In the outlier detection process, as soon as a notable deviation of a data point from the others is detected, it will be recognized as an outlier. This study seeks benefits from the well-known leverage value procedure for spotting an outlier^68^. Firstly, the standardized residual and the Hat and value corresponding to any input data were calculated. The following formulation was used for the determination of Hat matrix^37^:

13 $$H = X\left( {X^{T} X} \right)^{ - 1} X^{T}$$

In the abovementioned formula, *X* represents a matrix with a size of *N* × *P*, where the total number of data points is depicted by *N* and the number of inputs’ features is denoted by *P*. Additionally, an alarming leverage value was determined as follows^37^:

14 $$H^{*} = \frac{{3\left( {P + 1} \right)}}{N}$$

## Results and discussion

### Model development

For predicting the amount of CO_2_ absorbed on the surface of various MOFs, we developed various models using XGboost, LightGBM, Catboost and RF methods. In order to avoid overfitting, the grid search was used to optimize hyperparameters of models. Each model's grid search hyperparameters were different, and the importance of the hyperparameters was determined by theoretical and practical considerations. For each model, the following hyperparameters were employed. Table S1 shows the optimal values of the major hyperparameters, as well as the search intervals for the hyperparameters set for the machine learning models used in this study.

In Table S1, n_estimators represents the number of trees, subsample shows subsample ratio of columns when constructing a tree, C denotes a degree of importance that is given to misclassifications, max_depth is maximum depth of a tree, feature_fraction is parameters randomly selected in each iteration for building trees, learning_rate controls the impact of each tree on the final outcome.

### Model implementation and accuracy evaluation

**Figure 3:**
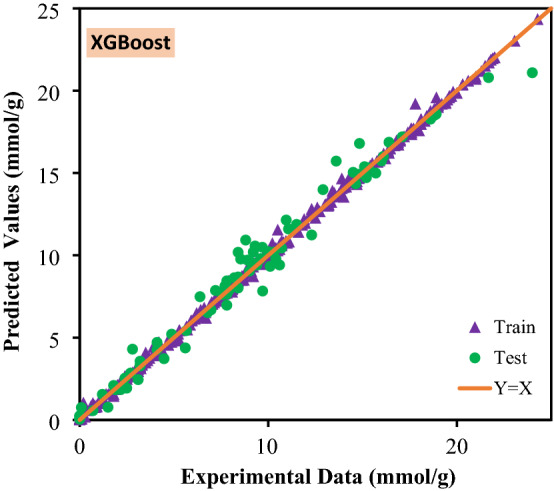
Crossplots of the proposed machine learning models in this study.

**Figure 4:**
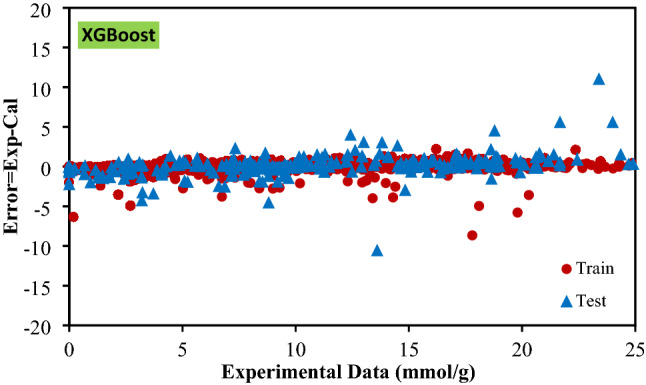
Error distribution plots of machine learning models for training and test sets.

**Figure 5:**
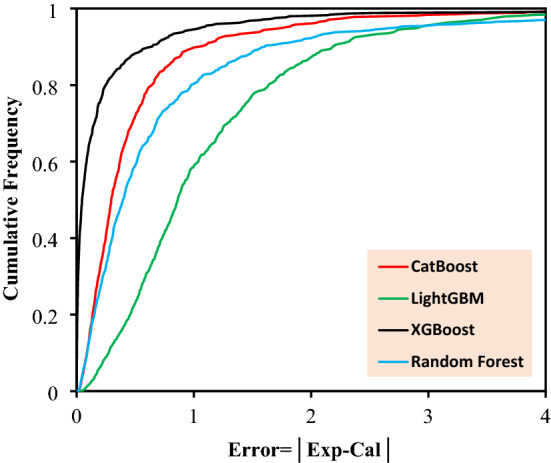
The cumulative frequency plot for the developed predictive models.

The most salient feature of each model is its accuracy and trustworthiness. In Fig. 3 and Fig. S3, the predicted CO_2_ adsorption capacity is illustrated versus the experimental data for the implemented models, in which the closer predicted data to the experimentally obtained data (forming a y = x line), the more accurate the model is. In the given figure, almost all models illustrate a slope very close to unity which prove that all of them can be reliable when anticipation of real data is needed. As an important statistical criterion, the calculated error corresponding to every data point is depicted in Fig. 4 and Fig. S4, which provide the readers with comprehensive information about the preciseness of the proposed models. Delineated in the given illustrations, data points fluctuate closely around the zero-error line which indicates that the models were developed well. According to this figure, the calculated error for RF, Catboost, LightGBM, and XGBoost are limited to ± 10, ± 8, ± 7, and ± 12.5, respectively. The cumulative frequency of errors of each developed model is illustrated in Fig. 5. As it can be seen, 95% of the data predicted by XGBoost model has an error less than 1%. Moreover, Catboost model could predict 89% data with errors less than 1%.

In Table 3, the accuracy of each model is reported based on statistical criteria. As it can be perceived from this table, having an R^2^ of 0.9992 and 0.9733 in the training and testing steps of modeling, the XGboost is the most trustworthy model followed closely by the Catboost model.

**Table 3:** Calculated statistical criteria for the developed models.

Statistical criteria		R^2^	RSME
XGBoost	Train	0.9992	0.2515
	Test	0.9733	1.1649
	Total	0.9955	0.5682
LightGBM	Train	0.9876	0.9819
	Test	0.9599	1.4245
	Total	0.9837	1.0853
CatBoost	Train	0.9660	1.6270
	Test	0.9653	1.3259
	Total	0.9659	1.5712
Random Forest	Train	0.9467	2.0364
	Test	0.9457	1.6600
	Total	0.9466	1.9667

Moreover, the calculated RSME for every model is given in Fig. S5 from which it is obvious that the XGBoost model is the most aureate model having an RSME of 0.568. Following closely, the LightGBM approach had the second lowest RSME value. According to both of RSME and R^2^ criteria, RF was determined as the poorest predicting model.

**Figure 6:**
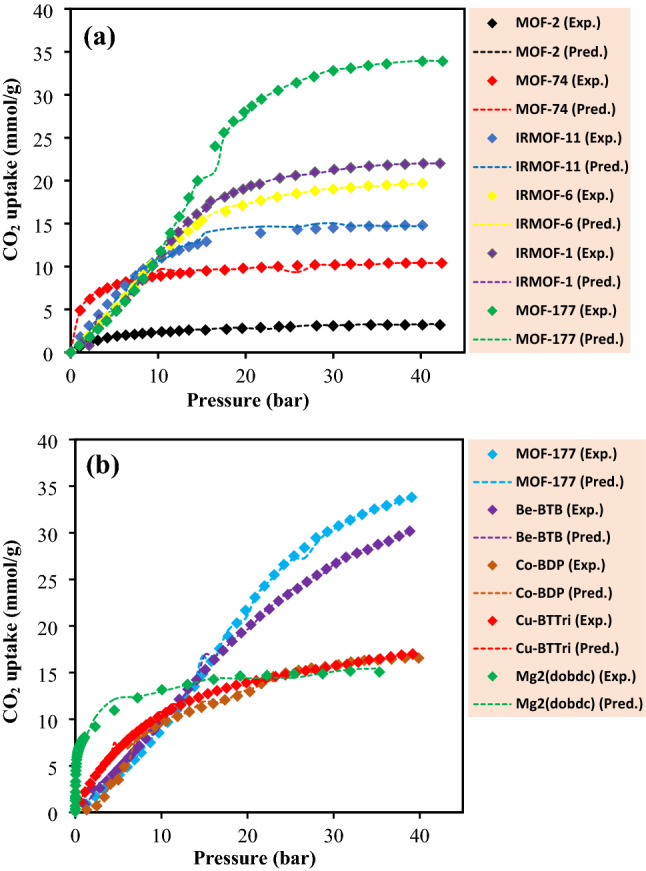
Comparison between experimental and predicted CO_2_ uptake capacities by XGBoost model for the investigated MOFs at different temperatures: (**a**) 298 K and (**b**) 313 K.

With increasing pressure, the saturation process of the MOFs’ pores will be facilitated according to the room-temperature isotherms. From these isotherms, it can be perceived that the uptake capacity of each MOF is qualitatively related to its surface area and among all types of assessed MOFs in the current study, the MOF-177, BeBTB, MOF-5 and PCN-11 showed outstanding and considerable CO_2_ adsorption capacity. As the pore sizes are greater and more efficient, the contribution of the pressure regime appearance gets more influence on the CO_2_ uptake capacity^69,70^. Comparison among all mentioned porous materials, the MOF-177 had a maximum capacity of 33.5 mmol/g, because of its high surface area (4508 m^2^/g) nearly twice of IRMOF-11. Figure 6a illustrates a detailed representation of uptake capacities in different pressures at 298 K for the investigated MOFs. Also, Fig. 6b draws a comparison between the CO_2_ adsorption efficiency of MOF-177 at 313 K and a range of other MOFs. It has also been discovered that the amount of CO_2_ adsorption on the MOF structure is directly related to the surface area and the polarity of the surface. As these features are higher, more CO_2_ will be absorbed and even in low pressures a high adsorption will be achieved using some MOFs like Cu-BTTri and Mg_2_(dobdc). Moreover, operating at a high pressure significantly affects the CO_2_ adsorption capacity.

**Figure 7:**
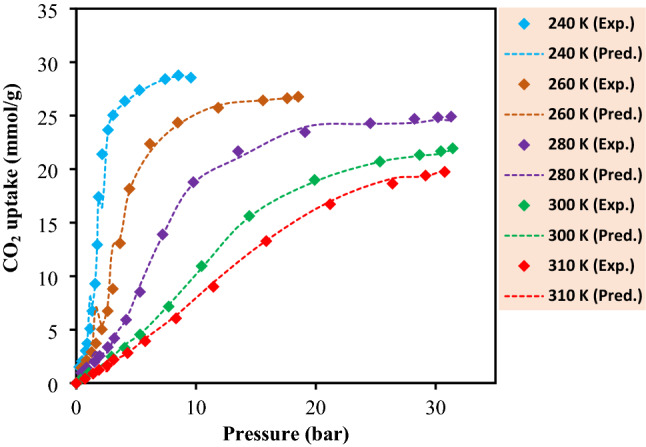
The effect of temperature on the CO_2_ adsorption capacity.

Regarding Co-BDP, a step-like appearance for the adsorption isotherm can be seen, which is potentially resulted from a phenomenon known as the gate opening. This event takes place due to the notable flexibility of the MOF’s framework^71,72^. This claim must not be interpreted as if the other MOFs are not efficient and practical in CO_2_ removal endeavors, but it means that MOF-177 has an excellent CO_2_ removal capacity at 35 bar^51^. Furthermore, as presented in Fig. 7, dependency of adsorption capacity on the operational temperature is considerable, and generally for MOF-5, the CO_2_ uptake capacity was higher at room temperature than its capacity at 313 K. Thus, temperature negatively affects the carbon dioxide adsorption efficiency. In other words, with increasing temperature, the adsorption capacity decreases.

**Figure 8:**
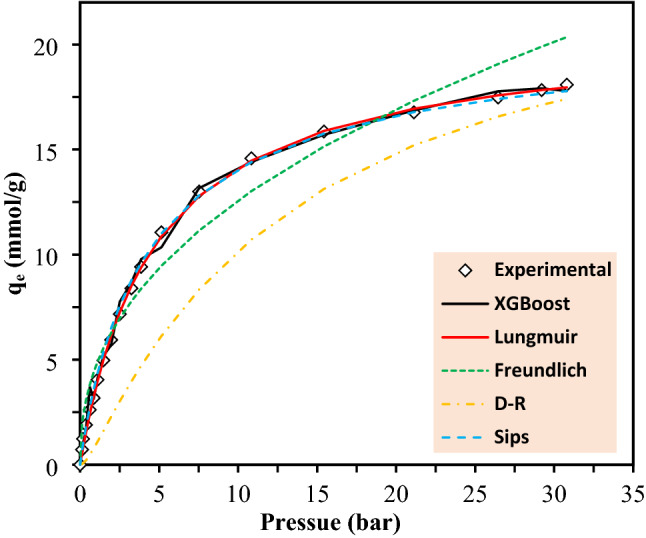
Comparison between different isotherms and XGBoost model for CO_2_ adsorption by PCN-11 at 313 K.

Ability of various models in prediction of CO_2_ adsorption capacity at low pressure ranges is shown in Fig. S6, in which the models were utilized to estimate CO_2_ removal efficiency of Mg_2_(dobdc) at 313 K. Among all models, the XGBoost and CatBoost were found to be the most trustable models. Moreover, this model can be successfully compared with different isotherm models. As illustrated in Fig. 8 and Fig. S7, XGBoost as the best model along with Langmuir, Freundlich, Dubinin-Radushkevitch (D-R) and Sips isotherm models were fitted with experimental data for PCN-11 and Mg-MOF-74. Figures show that both Langmuir and XGBoost models predicted the results well. Langmuir isotherm model showed well-fitting with correlation coefficient of 0.999 and 0.980 for PCN-11 and Mg-MOF-174, respectively, but totally the XGBoost models represented better fitting correlation with R^2^ values of 0.998 for both MOFs.

Microporous MOFs are new emerging generation of promising adsorbents which can drastically improve the CO_2_ removal efficiency due to their high selectivity ranges. Using these materials, a higher CO_2_ uptake capacity could be achieved^73^ specially between 5 and 40 bar which is appropriate for the separation of CO_2_ and H_2_^74,75^. In power plants in which coal is mainly consumed for generation of power, a wide range of pollutants will be emitted to the environment including carbon dioxide. As a highly efficient process, pre-combustion capture of CO_2_ can take place in the integrated gasification and combined cycle method. In this system, carbon dioxide could be separated from H_2_ and removed from the process^5,65,76^. Additionally, the intrinsic feature of the surface of these materials will result in a better and more powerful interaction between CO_2_ and the surface of the MOFs^77^. Due to their promising removal efficiency, many scientists have paid attention to integration of these materials into various industries including gas separation and purification processes in the petroleum industry specially when separation of CO_2_/N_2_^78^, CO_2_/CH_4_^71^, and O_2_/N_2_^79^ are desired. To successfully investigate the adsorption process, a profusion of experimental endeavors has been done and lot of isotherm models have been developed. Nevertheless, a range of drawbacks have prevented both of experimental works and theoretical approaches to successfully be used. Firstly, the designing and conducting experiments are time consuming and costly. Additionally, a wide range of various assumptions must be made to simplify the problem when the currently available adsorption isotherms are going to be utilized. Resultantly, not only a big inventory of data is needed, but also modeling process encounters errors from first the steps of the investigation. These errors will be exacerbated if a set of needed data is not accessible^31^. Therefore, application of cost-effective, time-saving, robust, and simple model is vital in the investigation and prediction of CO_2_ adsorption capacity by MOFs. The presented investigation has dealt with the adsorption of CO_2_, as one of the main causes of global warming issue^5^, on MOFs, which are known as very promising materials for CO_2_ removal, by applying some well-known soft computing techniques. The most outstanding feature of these models was this fact that no limitation for the development of these comprehensive models were needed to be applied. Therefore, being capable of handling a wide range of data, the implemented models have estimated the adsorption capacity at various pressures and temperatures accurately. However, the models’ performance and reliability are highly dependent on the selection of appropriate input parameters^1^. Additionally, successful application of smart models requires a large number of data points and excellent skills of programming.

**Figure 9:**
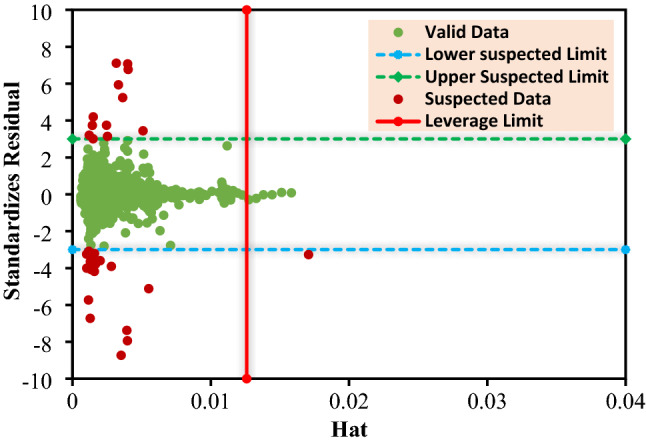
Williams plot for outlier detection of the proposed XGBoost model.

Based on the calculated Hat values, an area limited by standardized residuals of ± 3 and 0 ≤ H ≤ H^*^ is detected as the acceptable region. H* is determined for every model. The model is valid if majority of the data points are being located in the determined region. Figure 9 shows the Williams plot relating to the proposed XGBoost model as the best approach in this study. According to this figure, almost all data points were inside the acceptable region and just a few data points are located outside the desirable area. Thus, both developed model and experimental data are statistically valid.

### Sensitivity analysis

To investigate the impact of various parameters on the adsorption capacity of MOFs, a relevancy factor was defined as follows^80–82^:

15 $$r = \frac{{\mathop \sum \nolimits_{i = 1}^{N} \left( {X_{k,i} - \overline{X}_{k} } \right)\left( {y_{i} - \overline{y}} \right)}}{{\sqrt {\mathop \sum \nolimits_{i = 1}^{N} \left( {X_{k,i} - \overline{X}_{k} } \right)^{2} \mathop \sum \nolimits_{i = 1}^{N} \left( {y_{i} - \overline{y}} \right)^{2} } }}$$

in which *N*, *X*_*k,i*_, *Y*_*i*_, *X̅*_k_, *Y̅* stand for the number of assembled data points, the k^th^ parameter of i^th^ input value, i^th^ output data, mean of the kth input parameter, and the average of the outputs, respectively.

**Figure 10:**
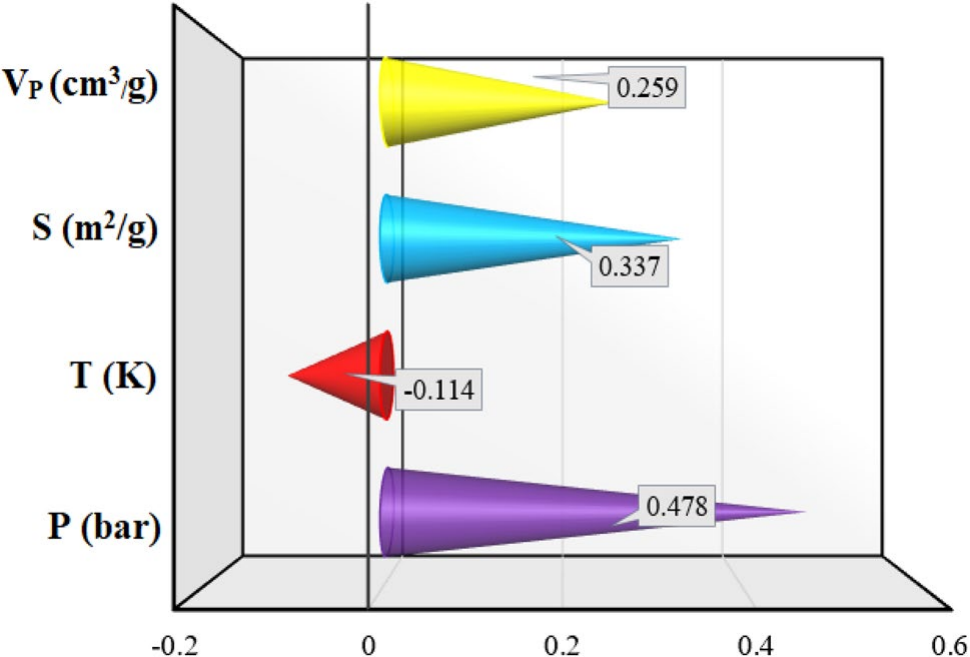
Evaluation of the input parameters' impact.

Laying between ± 1, the relevancy factor depicts how much an input parameter affects the CO_2_ removal capacity of the MOFs. As the absolute value of the relevancy factor corresponding to a specified parameter increases, the carbon dioxide adsorption capacity will change more dramatically with any change in that parameter. The calculated relevancy factor for each affecting parameter on the adsorption of CO_2_ on various MOFs is illustrated in Fig. 10. According to the calculated r values, among all investigated parameters, only temperature negatively impacts the adsorption capacity having an r = − 0.114. Furthermore, it was found that pressure and surface area of MOFs have the most and the second most significant effects on the CO_2_ adsorption capacities possessing “r” factors of 0.478 and 0.337, respectively. Pore volume also was found to have a positive impact on the CO_2_ adsorption capacity of MOFs as provides more adoption sites for CO_2_ molecules to be captured by them.

## Conclusions

The adsorption capacity of various MOFs for CO_2_ capture was modeled by development of robust models using 1191 data points. The data was used to train and test the XGBoost, LightGBM, CatBoost, and RF approaches and it was found that the XGBoost as the best fitting model and the Catboost as the second most trustworthy model predicted the CO_2_ adoptions more precisely than other methods. The R^2^ and RMSE values of 0.9955 and 0.5682, 0.9659 and 1.5712, 0.9837 and 1.0853, and 0.9466 and 1.9667 were obtained for XGBoost, CatBoost, LightGBM, and RF approaches, respectively. Additionally, it was found that all parameters except temperature have positive impact on the CO_2_ capture by MOFs and temperature had the smallest influence on the CO_2_ uptake capacity. It has also been discovered that the amount of CO_2_ adsorption on the MOF structure is directly related to the surface area and the polarity of the surface. As these features are higher, more CO_2_ will be absorbed and even at low pressures, a high adsorption could be achieved using some MOFs like Cu-BTTri and Mg_2_(dobdc). Langmuir isotherm model showed well-fitting with correlation coefficient of 0.999 and 0.980 for PCN-11 and Mg-MOF-174, respectively, but totally the XGBoost models represented better fitting correlation with R^2^ values of 0.998 for both MOFs. The obtained results of the current work could be beneficially utilized in the environmental studies such as carbon capture and gas separation and purification.

## Supplementary Information

Supplementary Information.
