# Supplementary material for: Modeling of CO2 adsorption capacity by porous metal organic frameworks using advanced decision tree-based models
Source: Sci Rep. 2021 Dec 28;11:24468. doi: 10.1038/s41598-021-04168-w (PMC8714819; doi:10.1038/s41598-021-04168-w)
Supplement: Supplementary file 1 — Supplementary Information. [file 41598_2021_4168_MOESM1_ESM.docx]

Table S1. Optimal features for implemented models.

| Optimum value/feature | Search range | Hyperparametr | Model |
| --- | --- | --- | --- |
| 80 | 1-100 | Max_depth | RandomForest |
| 3 | 2-5 | Max_features |  |
| 100 | 1−500 | n_estimators |  |
| True | True-False | bootstrap |  |
| 800 | 1−2000 | n_estimators | LightGBM |
| 0.1 | 0.01−0.9 | Learing_rate |  |
| 0.8 | 0.1−1 | Subsample |  |
| 12 | 1−14 | Max_depth |  |
| 400 | 1−2000 | n_estimators | XGBoost |
| 0.1 | 0.01−0.9 | Learing_rate |  |
| 0.9 | 0.1−1 | Subsample |  |
| 5 | 1−16 | Max_depth |  |
| 0.9 | 0.4−0.95 | Feature_fraction |  |
| 400 | 1−4000 | n_estimators | CatBoost |
| 0.15 | 0.01−0.9 | Learing_rate |  |
| 0.8 | 0.1−1 | Subsample |  |
| 5 | 1−16 | Max_depth |  |

**Figures**

**Fig. S1.** A schematic illustration of Leaf-wise growth of trees in LightGBM.

**Fig. S2.** A schematic illustration of random forest algorithm.‎

**Fig. S3.** Crossplots of the proposed machine learning models in this study.

**Fig. S4.** Error distribution plots of machine learning models for training and test sets.

**Fig. S5.** RSME values corresponding to each model.

**Fig. S6.** The prediction capability of the implemented models for Mg_2_(dobdc) at 313 K.

**Fig. S7.** Comparison between different isotherms and XGBoost model for CO_2_ adsorption by Mg-MOF-74.at 313 K.


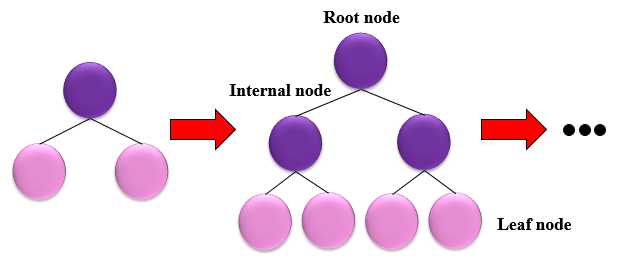


**Fig. S1.**


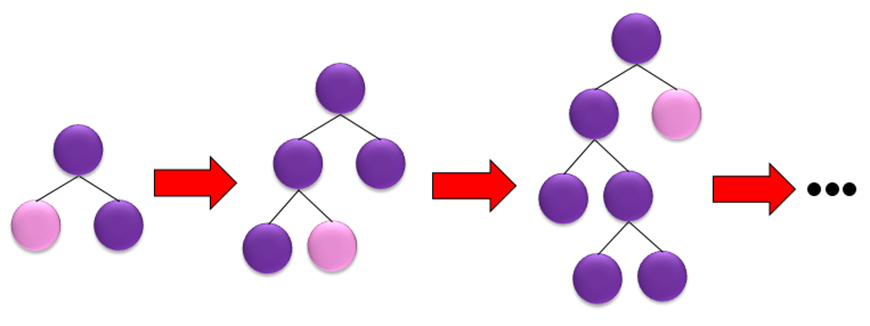


**Fig. S2.**

**Fig. S3.**

**Fig. S4.**

**Fig. S5.**

**Fig. S6.**

**Fig. S7.**
